# SG-APSIC1035: Prospective safety surveillance study of ACAM2000 smallpox vaccine in deployed military personnel

**DOI:** 10.1017/ash.2023.31

**Published:** 2023-03-16

**Authors:** Kevin Yeo, Daniel Gordon, Lori Perry, Ilfra Raymond-Loher, Nita Tati, Kevin Yeo, Grace Lin, Gina DiPietro, Alex Selmani, Michael Decker

**Affiliations:** Emergent BioSolutions, United Kingdom; Sanofi Pasteur Inc, Swiftwater, Pennsylvania, United States; Naval Health Research Centre, San Diego, California, United States; United Biosource Corp, Blue Bell, Pennsylvania, United States; Sanofi Pasteur, Swiftwater, Pennsylvania, United States; Emergent BioSolutions Inc, Gaithersburg, Maryland, United States; Emergent BioSolutions Inc, Gaithersburg, Maryland, United States; Emergent BioSolutions Inc, Gaithersburg, Maryland, United States; Sanofi Pasteur Inc, Swiftwater, Pennsylvania, United States; Sanofi Pasteur Inc, Swiftwater, Pennsylvania, United States

## Abstract

**Objectives:** We compared rates of myopericarditis adverse events and evaluated potential risk factors of development. We compared rates of dermatological–neurological adverse events (severe and serious) with other adverse events in a specific population of deployed US military personnel who received or did not receive ACAM2000 vaccine (ie, Vaccinia smallpox live vaccine). **Methods:** Up to 20,000 military personnel recipients were enrolled in a prospective observational cohort study: up to 15,000 ACAM2000 recipients in cohort 1 and up to 5,000 military personnel who were eligible for ACAM2000 vaccination but were not vaccinated due to recent vaccination or characteristics of their contacts in cohort 2. Enrollment was at a 3:1 ratio, respectively. Serum specimens and data were collected at the initial visit and 10 days later (cf, window of 6–17 days). Study participants with evidence, either clinical or laboratory, of possible myopericarditis were referred to a blinded independent review committee for further evaluation and adjudication. The primary analysis was logistic regression with adjudicated myopericarditis as the dependent variable and age, sex, race, and exercise regimen as the independent variables. **Results:** Initial data and serum specimens were obtained from 14,667 participants (cohort 1, N = 10,825; cohort 2, N = 3,842). According to protocol, 2 visits were completed by 12,110 participants (cohort 1, N = 8,945; cohort 2, N = 3,165), and 125 participants (cohort 1, N = 111; cohort 2, N = 14) were referred for myopericarditis adjudication, of whom 1 had confirmed myocarditis, 5 had suspected myocarditis, 1 had suspected pericarditis, and 54 (cohort 1, N = 44; cohort 2, N = 10) had subclinical myopericarditis. The unadjusted myopericarditis rates were 5.7 per 1,000 (95% CI, 4.3–7.5) for cohort 1 and 3.2 per 1,000 (95% CI, 1.7–5.8) for cohort 2. The unadjusted and adjusted odds ratios for myopericarditis for cohort 1 and cohort 2 were 1.8 (95% CI, 0.9–3.6) and 1.3 (95% CI, 0.6–2.6), respectively. At least 1 serious adverse event was experienced by 117 participants (1.1%) in cohort 1 and 13 (0.3%) in cohort 2. No serious and severe neurological or dermatological adverse events were reported. **Conclusions:** ACAM2000 vaccination was associated with a modest but nonsignificant increase in the risk of myopericarditis in this prudently screened, young and healthy service-member population. The adjusted OR was 1.3 and the unadjusted OR was 1.8. Overall, all but 7 cases were subclinical. Citation: Faix DJ, Gordon DM, Perry LN, et al. Prospective safety surveillance study of ACAM2000 smallpox vaccine in deploying military personnel. *Vaccine* 2020;38:7323–7330.

